# [Retracted] Silica nanoparticles induce cardiomyocyte apoptosis via the mitochondrial pathway in rats following intratracheal instillation

**DOI:** 10.3892/ijmm.2026.5728

**Published:** 2026-01-05

**Authors:** Zhongjun Du, Shangya Chen, Guanqun Cui, Ye Yang, Enguo Zhang, Qiang Wang, Martin F. Lavin, Abrey J. Yeo, Cunxiang Bo, Yu Zhang, Chao Li, Xiaoshan Liu, Xu Yang, Cheng Peng, Hua Shao

Int J Mol Med 43: 1229-1240, 2019; DOI: 10.3892/ijmm.2018.4045

Following the publication of this article, a concerned reader drew to the Editor's attention that the image showing silica nanoparticles in Fig. 1 on p. 1232 had also been used to show the same data in another paper published by the same research group in *International Journal of Molecular Medicine*. Upon performing a separate investigation of the data in this paper in the Editorial Office, it also came to light that, concerning the immunohistochemical images shown in Fig. 8A on p. 1236, five pairs of data panels out of a total of 12 panels included in this figure contained overlapping sections of data, occasionally in different orientations with respect to other panels, such that data which were intended to show the results from differently performed experiments had apparently been derived from a smaller number of original sources.

Given the large number of panels in this figure that were revealed to have overlapping sections, the Editor of *International Journal of Molecular Medicine* has decided that this article should be retracted from the Journal. The authors were asked for an explanation to account for these concerns, but the Editorial Office did not receive a reply. The Editor apologizes to the readership for any inconvenience caused.

